# Survival of *Xanthomonas vasicola* pv. *vasculorum* in Soil and in Corn Crop Residues under the Humid Subtropical Climate of Southern Brazil

**DOI:** 10.3390/life14070825

**Published:** 2024-06-28

**Authors:** Talita Vigo Longhi, Renata Rodrigues Robaina, Rui Pereira Leite, Maria Isabel Balbi-Peña

**Affiliations:** 1 Instituto de Desenvolvimento Rural do Paraná—IAPAR/Emater (IDR-Paraná), km 375 Celso Garcia Cid Road, Londrina 86047-902, Brazil; talitalonghi@hotmail.com (T.V.L.); robainarr@yahoo.com.br (R.R.R.); ruileite@idr.pr.gov.br (R.P.L.J.); 2 Departamento de Agronomia, Universidade Estadual de Londrina (UEL), km 380 Celso Garcia Cid Road, Londrina 86057-970, Brazil

**Keywords:** bacterial leaf streak of corn, crop residues, time of survival, *Zea mays*

## Abstract

Bacterial leaf streak caused *by Xanthomonas vasicola* pv. *vasculorum* (Xvv) is an emerging disease in several corn-producing regions around the world. In Brazil, there is a lack of information on the survival of this bacterium in soil and crop residues. Thus, the objective of this study was to determine the survival of Xvv in soil and also in infected corn crop residues under the humid subtropical climate of southern Brazil. The survival of Xvv in soil was initially investigated in sandy and clayey soils maintained at 20, 25 and 30 °C under controlled conditions. The survival of the bacterium under field conditions was studied in artificially infested clayey soil. The survival of Xvv in corn crop residues was investigated in infected residues maintained on the soil surface or buried in the soil at 20 cm deep. Under controlled conditions, regardless of the type of soil, the bacterium survived longer at 20 °C than at higher temperatures. The bacterium survived for 40 days in clayey soil kept at 20 °C and four days in sandy soil maintained at 30 °C. Under field conditions, the survival of Xvv in the soil was only for 48 h and in infected corn crop residues for up to 15 days in the samples maintained on the soil surface. In samples of infected corn residues buried in the soil, the bacterium was only detected at the time the experiment was set up. In general, the results obtained in this study revealed that Xvv survives for a short period of time in soil and in infected corn crop residues under humid subtropical conditions. Therefore, soil and corn residues may not be highly important sources of primary inoculum for the development of bacterial leaf streak on corn crops under these conditions.

## 1. Introduction

Bacterial leaf streaks occur in a wide range of cereals and have the potential to cause significant yield and quality losses, threatening food security and social stability. These diseases are caused by bacteria of different species of the genus *Xanthomonas*, including *X. translucens* on several cereals, *X. vasicola* on corn and sorghum, and *X. oryzae* on rice [1]. The bacterial leaf streak of corn, caused by *Xanthomonas vasicola* pv. *vasculorum* (Xvv), was first described in South Africa in 1949 [2] and remained for decades without being reported in other corn-producing regions around the world. However, it has recently been introduced in important producing regions of some countries in North and South Americas, including the United States, Argentina and Brazil [3,4,5,6,7,8]. 

As a poorly studied disease, there is a lack of information on several aspects of bacterial leaf streak, including the survival capability of the bacterium in soil and in corn plant residues. However, this information is extremely important for the establishment of efficient measures for the prevention and management of bacterial leaf streak of corn. For instance, the survival of Xvv in the corn crop off-season, even in low populations, may be sufficient to guarantee the occurrence of new epidemic cycles of the disease [9,10,11,12]. Ortiz Castro [13] has already demonstrated that corn crop residues maintained on the soil surface can be important sources of primary inoculum for the development of the disease in continuous cereal production systems under temperate climate conditions.

Plant pathogenic bacteria can generally survive in seeds or organs of vegetative propagation, in infected plant tissues, in crop residues, in the soil, as epiphytic residents, or in volunteer plants of the crop itself or other host plants, cultivated or invasive [9,14,15]. The survival period of plant pathogenic bacteria in the soil usually depends on several environmental factors such as temperature and humidity, and the physical, chemical and biological characteristics of the soil [16,17]. The survival in the soil and crop residues is not only a concern for the management of bacterial leaf streak of corn, but has also been a concern for other diseases caused by *Xanthomonas* spp. [18,19,20]. Therefore, studies on the survival capability of Xvv in the soil or in association with corn crop residues may allow the adoption of more appropriate practices for the management of bacterial leaf streak in Brazil and in other corn-producing countries with similar climatic conditions. Further, there are still no published studies on the survival of Xvv bacterium under subtropical conditions. 

In the USA, studies were carried out under field conditions to determine the survival of Xvv in naturally infected corn plant residues maintained on the surface or buried in the soil [13]. Although it was not possible to recover viable Xvv cells, bacterial DNA was detected by real-time PCR (qRT-PCR) for up to six months in residues maintained in the field. However, the technique used for detection, qRT-PCR, did not allow determining if the DNA detected was from viable Xvv cells. It is worth noting that the qRT-PCR technique has also been used to improve the detection of other plant pathogenic *Xanthomonas* in soil, enabling determining the presence of *X. campestris* pv. *campestris* in this environment for up to two years [18].

The present study aimed to evaluate the survival of Xvv in soil and in corn residues infected with the bacterium under controlled and field conditions. This study considered (i) the influence of temperature and soil type on the survival of Xvv under controlled conditions, (ii) the survival of Xvv in soil under field conditions and (iii) the survival of Xvv in corn crop residues infected with the bacterium maintained on the surface or buried in the soil under field conditions.

## 2. Materials and Methods

### 2.1. Obtaining and Maintaining RL1 Xanthomonas vasicola pv. vasculorum Rifampicin-Resistant Mutant (RL1^Rif+^)

The RL1 strain of Xvv used in this study belongs to the bacterial collection of the Bacteriology Laboratory of the Institute of Rural Development of Paraná—IAPAR/Emater (IDR-Paraná), Londrina, PR, Brazil. The identity of this bacterial strain was confirmed by PCR using specific oligonucleotide primers for Xvv, Xvv3_F (5′-CAAGCAGAGCATGGCAAAC-3′) and Xvv3_R (5′-CACGTAGAACCGGTCTTT GG-3′), which amplify a 207 bp fragment of the bacterial genome [21]. The RL1 rifampicin-resistant mutant (RL1^Rif+^) was obtained by plating a bacterial suspension on nutrient agar (NA) culture medium (5.0 g of peptone, 3.0 g of meat extract, 15.0 g of agar and 1000 mL of distilled water) [22], supplemented with 100 µg mL^−1^ of rifampicin (INLAB Confiança, São Paulo, Brazil), followed by incubation at 28 °C for 72 h. The RL1^Rif+^ mutant was stored in phosphate buffer at room temperature.

The pathogenicity of the RL1^Rif+^ bacterium was confirmed by inoculation on plants of the IPR 164 corn variety, susceptible to bacterial leaf streak, maintained under greenhouse conditions. Seeds were sown in 5 L pots (16 cm high × 21 cm in diameter) containing a mixture of soil, sand and manure (3:1:1) maintained in a semi-climatized greenhouse with temperature ranging between 25 and 30 °C, at the Londrina Research Station of the IDR-Paraná, Londrina, PR, Brazil. The plants were inoculated by spraying a cell suspension of the RL1^Rif+^ bacterium adjusted to a concentration of 10^8^ UFC mL^−1^ (DO_600_ nm = 0.1). The inoculated corn plants were kept in the same greenhouse under high-humidity conditions for 24 h before and 24 h after inoculation.

### 2.2. Survival of Xanthomonas vasicola pv. vasculorum in Soil as Free Cells under Controlled Conditions

The soils used in the experiments were collected in two arable areas located in the North and Northwest regions of the state of Paraná, Brazil. The clayey soil was collected at the Londrina Research Station of the IDR-Paraná, located in Londrina, PR (latitude of 23°21′30″ S; longitude of 51°10′17″ W; and altitude of 585 m), and the sandy soil was collected at the Technological Development Unit (UDT) of the Cooperativa Agroindustrial Cocamar, located in the municipality of Guairaçá, PR (latitude of 22°56′04″ S; longitude of 52°41′08″ W; and altitude of 518 m) [23]. These places were selected because the soils have distinct geological origins and differences in particle size distribution and, consequently, have different chemical and physical attributes. The clayey soil was classified as *Distroferric Red Latosol* (Rhodic Hapldox), and the sandy soil as *Dystrophic Yellow Oxisol* (Typic Haplustox) [24]. The climate of both regions is classified as humid subtropical (Cfa), according to the Köppen–Geiger climate classification, with annual maximum and minimum temperatures of 27.1 and 16.1 °C for Londrina, and 29.1 and 17.1 °C for Guairaçá [23].

The soils were collected at a depth of 20 cm in areas with no recent history of corn cultivation. The soil analysis was carried out in the Soil and Plant Tissue Analysis Laboratory at the IDR-Paraná, Londrina, PR, Brazil. The determination of the chemical attributes of the soils was carried out according to the reference procedure used by the IDR-Paraná and described by Pavan et al. [25]. 

The soils were dried at room temperature for 12 days and sieved through a 2.0 mm mesh before the experiments were set up to standardize all repetitions of the treatments in relation to the distribution of aggregates in the same type of soil and also between different soils. Aliquots of 150 g of each soil were placed in 400 mL polystyrene cups. The clayey and the sandy soils were infested with a suspension of the Xvv RL1^Rif+^ bacterium at a concentration of 10^8^ UFC mL^−1^ saturating each soil up to their field capacity. The cups were closed with aluminum foil and kept at temperatures of 20, 25 and 30 °C in a Biochemistry Oxygen Demand (B.O.D.)-type incubator (411 FPD, Labstore, Vargem Grande Paulista, SP, Brazil). Soil moisture correction was carried out when needed according to a previously described protocol [26]. The experimental design was completely randomized in a 2 × 3 factorial scheme (two types of soils x three temperatures) with four replications.

For the evaluation of the survival of Xvv, four cups were sampled for each type of soil and temperature, and the samples were processed individually. After the samples were removed, the cups were returned to the incubator at the corresponding temperature.

To determine the Xvv population, 10 g aliquots of soil from each sample were transferred to an Erlenmeyer containing 90 mL of autoclaved phosphate buffer 0.005 M (1.73 g K_2_HPO_4_, 1.36 g KH_2_PO_4_, 2000 mL of distilled water, pH 7.0). The samples were shaken at 250 rpm for 30 min, and kept to rest another 30 min for the soil to sediment. Aliquots of 1 mL of the supernatant were collected, diluted, and 100 μL of each dilution was plated on NA medium [22] supplemented with rifampicin and cycloheximide (Sigma-Aldrich, Saint Louis, MO, USA), both at the concentration of 100 µg mL^−1^. The plates were kept at 28 °C and bacterial colonies characteristics of the RL1^Rif+^ bacterium were counted after 72 h. 

At each evaluation period, 5 to 10 bacterial colonies counted for each sample had their identity confirmed by PCR test [21]. For total DNA extraction, a suspension of 10^8^ CFU mL^−1^ of each bacterial culture was subjected to a water bath at 100 °C for 5 min, followed by rapid cooling on ice. 

The PCR reaction was carried out in a final volume of 25 μL, containing: 1.0 μL of each primer (0.5 mM), 1.0 μL of dNTP (5 mM), 0.8 μL of MgCl_2_ (50 mM), 2.5 μL of buffer (1X), 0.2 μL of Taq DNA Polymerase Recombinant (5 U μL^−1^), 17.5 μL of ultrapure water and 1 μL of total DNA. A total of 30 cycles of amplification was carried out in a Veriti™ 96-Well thermocycler (Thermo Fisher Scientifc, Marsiling Industrial Estate Road 3, Singapore), comprising denaturation at 94 °C for 30 s, annealing at 55 °C for 30 s and extension at 72 °C for 1 min, with a final elongation step at 72 °C for 10 min and maintained at 4 °C.

In both experiments, samples were taken before soil infestation and 0, 1, 2, 4, 8, 12, 15, 20, 25, 30, 35, 40 and 45 days after infestation (d.a.i) with the RL1^Rif+^ bacterium.

### 2.3. Survival of Xanthomonas vasicola pv. vasculorum in Soil as Free Cells under Field Conditions

Four experiments were conducted in an experimental area of clayey soil not previously cultivated with corn at the Londrina Research Station of the IDR-Paraná, Londrina, PR, Brazil (latitude of 23°21′30″ S; longitude of 51°10′17″ W; and altitude of 585 m), during 2020 and 2021. 

Meteorological data, including maximum and minimum temperatures, relative air humidity and rainfall during the experimental periods, were obtained from the Meteorological Station of Londrina of the IDR-Paraná, located at a distance of approximately 100 m from the experimental area. Soil moisture data were collected using the ML3 Theta Probe device (Low Road, Burwell, Cambridge, UK), once a day in the afternoon. All weeds in the experimental area were removed before each experiment was set up.

Wooden frames measuring 30.0 × 30.0 × 8.0 cm in width, length and height, respectively, were distributed 60 cm apart in the experimental area to delimit the experimental plots. For each experiment, six wooden frames were used. The soil inside each frame was artificially infested with 1 L of a suspension of the Xvv RL1^Rif+^ bacterium at a concentration of 10^8^ CFU mL^−1^.

Sampling was carried out using a manual auger, collecting soil in each plot up to 5 cm deep. For each assessment, three soil samples were removed from each plot to form a composite sample. Samples were collected at 0, 1, 2, 4 and 6 days after soil infestation. The survival of the bacterium was examined until non-detection of Xvv RL1^Rif+^ bacterium in at least two subsequent samplings. The period of survival of Xvv was considered as the last sampling with presence of the bacterium. 

To recover Xvv, 10 g of soil of each composite sample were transferred to 250 mL Erlenmeyer flasks containing 90 mL of sterilized phosphate-buffered saline solution (0.01 M PBS, pH 7.0). The Erlenmeyer flasks were shaken at 200 rpm for 30 min, followed by resting for 30 min for the soil to sediment [19]. The supernatant was recovered and diluted to 10^−3^, and 100 μL of the supernatant of each dilution was plated in triplicate on NA medium supplemented with 100 μg mL^−1^ of rifampicin and 100 μg mL^−1^ of cycloheximide. The plates were kept at 28 °C for 72 h and colonies identical to those of the Xvv RL1^Rif+^ bacterium were quantified. From each sampling performed, 5 to 10 bacterial colonies counted were purified and had their identity confirmed by PCR, as previously described. 

### 2.4. Survival of Xanthomonas vasicola pv. vasculorum in Infected Corn Crop Residues

Two experiments were conducted at the Londrina Research Station of the IDR-Paraná, Londrina, PR, Brazil. The first experiment was carried out in the winter, from 24 July through 22 September 2020, and the second experiment during the spring, from 24 November through 22 December 2020. The experimental area was previously cultivated with oat, and was kept free of weeds by manual removal. Temperature and rainfall data were obtained from the Meteorological Station of Londrina of the IDR-Paraná, located at 100 m from the experimental area.

To obtain crop residues infected with the bacterium, plants of the IPR 164 variety produced under controlled conditions in 5 L pots (16 cm high × 21 cm in diameter) were inoculated approximately 12 days after emergence at the V3 phenological stage. After the development of bacterial leaf streak symptoms, approximately 15 days after inoculation, symptomatic leaves were collected, dried at room temperature for seven days, packed in paper bags and stored in a cold room at a temperature of 8 to 12 °C.

After four months of storage, samples containing eight grams of dry leaves were transferred to nylon bags measuring 20 × 30 cm. The bags were tied to 60 cm long wooden stakes, with 60 bags kept on the soil surface and the other 60 bags buried in the soil at a depth of 20 cm. The survival of the bacterium was examined in the first experiment at 0, 15, 30, 45 and 60 days, and in the second experiment at 0, 2, 4, 8, 12, 15, 20, 25 and 30 days after the set up of the experiment. Three bags were sampled from the surface of the soil and three from the ones kept 20 cm deep in the soil at each time. 

Samples of the plant material were taken from the field, weighed and crushed in an analytical mill (IKA A11 basic, Staufen, Germany). Sterilized 0.01 M phosphate buffer saline solution, pH 7, was added at the rate of 10 mL of solution per gram of plant material and the samples were subjected to ultrasonication (Thornton T50, Vinhedo, SP, Brazil) for 20 min. The suspension obtained from each sample was filtered through a double layer of gauze and the liquid was diluted to 10^−3^. Aliquots of 100 uL of each dilution were plated on NA culture medium supplemented with 100 μg mL^−1^ rifampicin and 100 μg mL^−1^ cycloheximide and maintained at 28 °C for 72 h. At each sampling, 5 to 10 bacterial colonies with characteristics similar to those of the Xvv RL1^Rif+^ bacterium were examined for further confirmation of the identity by PCR, as previously described.

### 2.5. Statistical Analysis

Bacterial population data were expressed in log_10_ UFC g soil^−1^ and subjected to analysis of variance (*p* < 0.05). The assumptions of normality of errors and homogeneity of variances were tested by the Shapiro–Wilk [27] and Levene tests [28], respectively, at 5% probability. When a significant effect was found, polynomial, linear or bisegmented regression analysis was performed using the “Segmented” package of the R software [29]. Survival analysis was also performed using the Kaplan–Meier estimator [30]. Treatments were compared using the log-rank test [31]. Analysis was carried out using the R package “Survival” [29].

## 3. Results

### 3.1. Survival of Xanthomonas vasicola pv. vasculorum in Soil under Controlled Conditions

**Figure 1:**
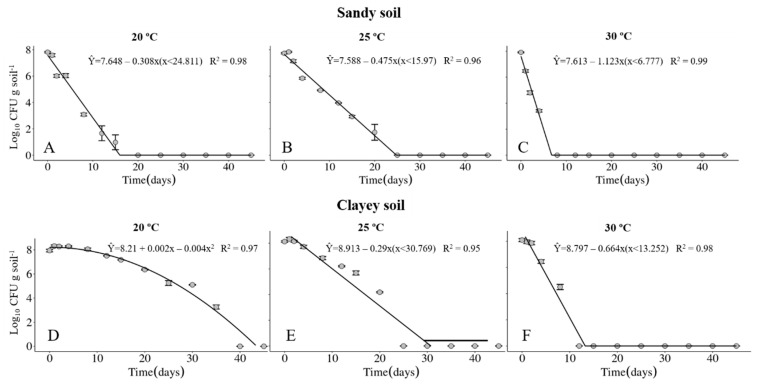
Survival of *Xanthomonas vasicola* pv. *vasculorum* RL1^Rif+^ (Log_10_ CFU.g solo^−1^) in two soil types under three different temperatures in the first experiment. (**A**–**C**), sandy soil; (**D**–**F**), clayey soil; (**A**,**D**), 20 °C; B and E, 25 °C; (**C**,**F**), 30 °C. Bars indicate the mean ± standard error.

**Figure 2:**
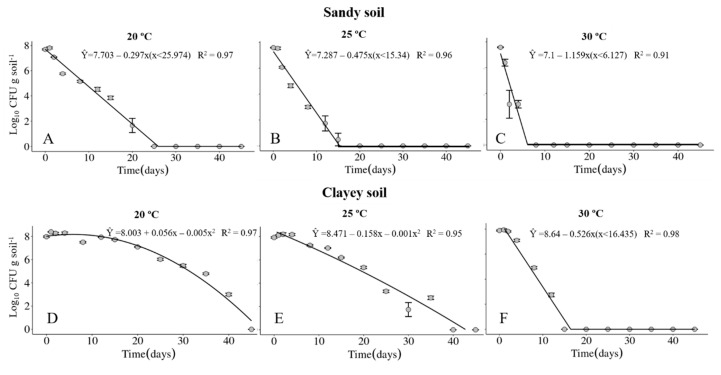
Survival of *Xanthomonas vasicola* pv. *vasculorum* RL1^Rif+^ (Log_10_ CFU.g solo^−1^) in two soil types under three different temperatures in the second experiment. (**A**–**C**), sandy soil; (**D**–**F**), clayey soil; (**A**,**D**), 20 °C; (**B**,**E**), 25 °C; (**C**,**F**), 30 °C. Bars indicate the mean ± standard error.

The survival of the Xvv RL1^Rif+^ was investigated in sandy and clayey soils, maintained at temperatures of 20, 25 and 30 °C. The physical and chemical properties of the soils are described in Table 1 and Table 2, respectively. Regardless of the type of soil, the maximum period of survival of the Xvv bacterium was at the temperature of 20° C (Figure 1A,D and Figure 2A,D). In clayey soil, the survival time of XvvRL1^Rif+^ was up to 35 and 40 days in the first and second experiments, respectively, at the temperature of 20 °C (Figure 1D and Figure 2D). On the other hand, the survival period of the bacterium in sandy soil in both experiments was up to 20 days, also at the temperature of 20 °C (Figure 1A and Figure 2A).

**Figure 3:**
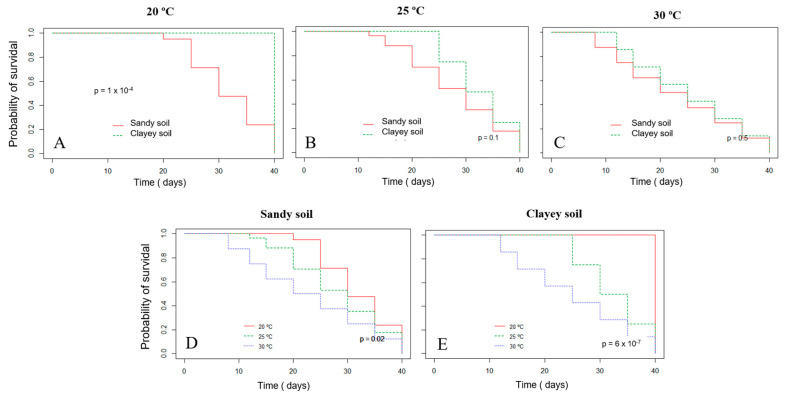
Survival probability of *Xanthomonas vasicola* pv. *vasculorum* RL1^Rif+^ in two types of soil under different temperatures based on the Kaplan−Meyer analysis in the first experiment. (**A**), clayey and sandy soils; (**A**), 20 °C; (**B**) 25 °C; (**C**) 30 °C; (**D**), sandy soil at different temperatures; (**E**) clayey soil at different temperatures. The log-rank test was used for comparisons.

**Figure 4:**
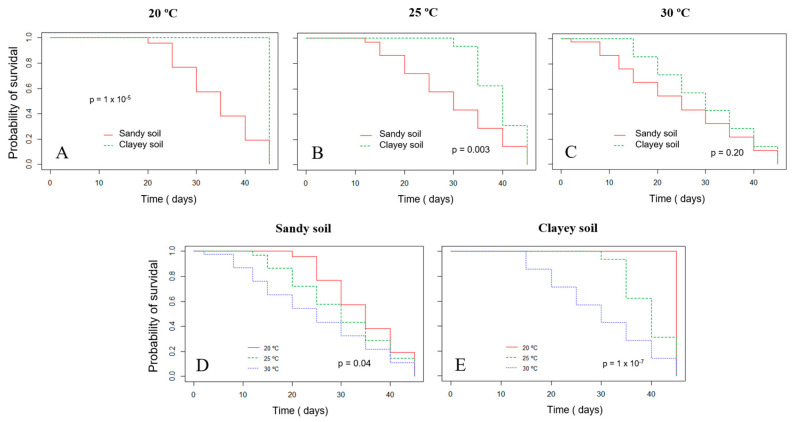
Survival probability of *Xanthomonas vasicola* pv. *vasculorum* RL1^Rif+^ in two types of soil under different temperatures based on the Kaplan–Meyer analysis in the second experiment. (**A**–**C**), clayey and sandy soils; (**A**) 20 °C; (**B**) 25 °C; (**C**) 30 °C; (**D**) sandy soil at different temperatures; (**E**) clayey soil at different temperatures. The log-rank test was used for comparisons.

The survival probability of Xvv RL1^Rif+^ assessed by the Kaplan–Meier analysis revealed that there was a significant difference in both experiments for the survival in sandy and clayey soils at the temperature of 20 °C (Figure 3A and Figure 4A). Thus, there was a higher probability of the bacterium to survive for a longer period in clayey soil maintained at a temperature of 20 °C as compared to the survival in the sandy soil. In the second experiment, there was also significant difference in the survival in sandy and clayey soils (*p*-value = 0.003) at 25 °C (Figure 4B). The survival probability of Xvv in both sandy and clayey soils was significantly higher at a temperature of 20 °C as compared to the survival at the temperatures of 25 and 30 °C (Figure 3D,E and Figure 4D,E). 

### 3.2. Survival of Xanthomonas vasicola pv. vasculorum in Soil under Field Conditions

**Figure 5:**
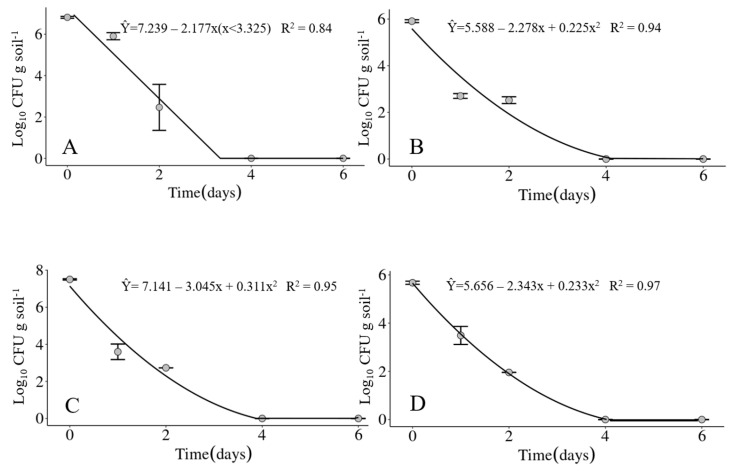
Survival of *Xanthomonas vasicola* pv. *vasculorum* RL1^Rif+^ in soil under field conditions in experiments carried out at the Londrina Research Station of the IDR-Paraná, Londrina, PR, Brazil, in the years of 2020 and 2021. (**A**) Experiment I; (**B**) Experiment II; (**C**) Experiment III; (**D**) Experiment IV.

The mean of daily average air temperatures of the four experimental periods did not vary much, ranging from 20.2 °C in experiment IV up to 23 °C in experiment III. The minimum temperatures ranged between 11.8 (Exp. IV) up to 20.1 °C (Exp. III) and the maximum between 22.0 (Exp. IV) and 34.2 °C (Exp. III), with the environmental conditions being the coldest during the experiment IV and the hottest in the experiment III. Accumulated precipitation varied from zero in experiment IV up to 45.4 mm in experiment II. Soil moisture reflected the precipitation during the experimental periods, with 7% on the average over the six days of the experiment IV compared to 59% on the average over the same period in experiment II. The average relative humidity of each experimental period was not sensitive to differences in accumulated precipitation in the same periods (Table 3). Considering the four experiments carried out in 2020 and 2021, the bacterium was detected only up to two days in the soil under field conditions. However, the maximum survival time of the bacterium may not have been determined very precisely as samplings were carried out only at 1, 2, 4 and 6 days after soil infestation. The bacterium could have survived until the third day (Figure 5A–D).

### 3.3. Survival of Xanthomonas vasicola pv. vasculorum in Infected Corn Crop Residues

**Figure 6:**
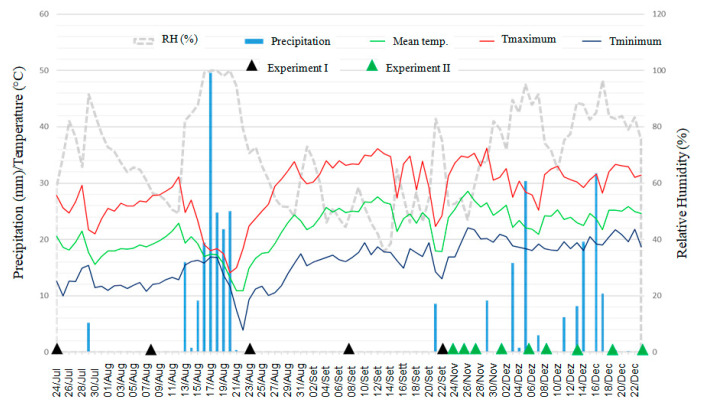
Meteorological data of precipitation (mm), minimum, maximum and average temperatures (°C), and relative humidity (%) at the IDR-Paraná Londrina Research Station, Londrina, PR, Brazil, during the period from 24 July through 22 December 2020. The black arrows indicate the samples date for evaluation of Xvv in the first experiment and the green arrows indicate the samples date in the second experiment.

During the first 15 days after the experiment was set, accumulated precipitation ranged from 5.2 mm for the first experiment up to 60 mm for the second experiment (Figure 6). The average maximum and minimum temperatures in the first 15 days of the experiments were, respectively, 25.7 °C and 12.1 °C for the first experiment and 31.4 °C and 19.4 °C for the second experiment (Figure 6).

**Figure 7:**
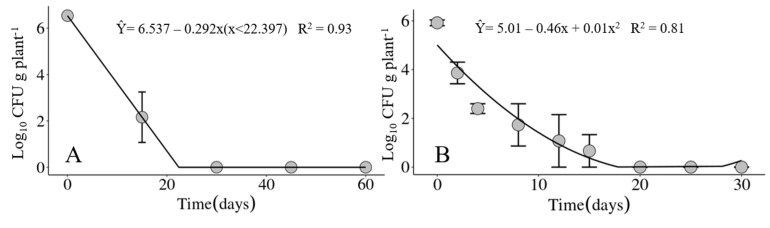
Population dynamics of *Xanthomonas vasicola* pv. *vasculorum* RL1^Rif+^ in infected corn crop residues maintained on the soil surface under field conditions in (**A**) the first experiment from 24 July through 22 September 2020 and in (**B**) the second experiment from 24 November through 22 December 2020, carried out at the IDR-Paraná Londrina Research Station, Londrina, PR, Brazil.

In the two experiments carried out in different seasons of the year, winter and spring, the maximum recovery period for the Xvv bacterium was 15 days in infected corn crop residues maintained on the soil surface (Figure 7). For the crop residues buried in the soil at a depth of 20 cm, the bacterium was only recovered at the time the experiments were set up.

## 4. Discussion

The survival of Xvv as free cells was investigated in soil under controlled and field conditions, and in infected corn residues obtained from plants with bacterial leaf streak symptoms. This is probably the first study on the survival of Xvv carried out under the humid subtropical conditions of southern Brazil. The Xvv RL1^Rif+^ mutant, obtained in this study, showed stability for resistance to the antibiotic rifampicin and the pathogenicity to corn plants was the same as that of the wild-type RL1 strain. These characteristics were important as the mutant could be used in the studies of the bacterial survival in soil and in corn plant residues. It should be pointed out that the presence of bacteria resistant to certain antibiotics such as rifampicin is uncommon under natural field conditions [32]. Thus, the use of this marker in Xvv turned possible to monitor the population dynamics of the bacterium in the present study, as soil samples from the field normally have a large microbial community.

The survival of the Xvv bacterium as free cells in soil was influenced by the temperature and soil type. Further, the survival period of the bacterium ranged from four up to 40 days in soil under controlled conditions, with the longest survival period in clayey soil maintained at 20 °C. Temperatures of approximately 20 °C favored the survival of Xvv in the soil when compared to higher temperatures, as for instance 30 °C. Similar results were obtained for *Xanthomonas campestris* pv. *campestris* that survived in soil for 14 days at 20 °C, but only four days when maintained at 30 °C [33]. 

Despite differences in the amount of bacterial cells used in the artificial infestation of the two types of soil, the mineralogical characteristics of the soils probably played a major role in the survival capability of Xvv in the clayey and sandy soils. The difference in carbon content between the two types of soil (almost 3.5-fold higher in clayey soil) could also played a role on the longer survival of the bacterium in clayey soil. Furthermore, in clayey soils, most bacteria could survive for longer periods, as they would be protected within clay aggregates [34]. Under these conditions, a protective envelope may be formed around the bacterial cells. This protective envelope would reduce water losses and could protect the bacterial cells during periods of soil desiccation and rehydration [35] and would also provide protection against antagonists [34]. In contrast, sandy soils would have a lower capability to provide such a protection to the bacterial cells due to the low formation of aggregates, compared to clayey soils [34]. In studies on the survival in different soil types, *Curtobacterium flaccumfaciens* pv. *flaccumfaciens* survived longer in clayey soils than in medium textured soils [19,36].

Soil pH can also interfere directly on the survival of plant pathogenic bacteria [37]. Usually, the optimum pH for bacterial multiplication ranges between 6.5 and 7.5 [38]. However, there are still no studies on the ideal pH for Xvv multiplication. In the present study, the bacterium survived in clayey soil with pH 5.0 for up to 40 days, while in sandy soil with pH 4.5, Xvv survived for up to 20 days. Although sandy soils may be less conducive for bacterial survival, these results suggest that the pH may also have influenced the survival of Xvv. Silva Júnior et al. (2020) [33] found that the survival of *X. campestris* pv. *campestris* in medium textured soils with pH 4.2 was only 10 days, while in clayey soil with pH 5.9 the bacterium could survive for 24 days. The authors concluded that higher pH probably favored the survival of *X. campestris* pv. *campestris.* As the survival of Xvv under controlled conditions was longer in clayey soil, a field area with this type of soil was chosen to carry out the experiments under field conditions. However, Xvv showed limited survival capability as free cells in the soil under these conditions. The bacterium survived for only 48 h in all experiments carried out in the field, even though a bacterial suspension at a high concentration (10^8^ UFC mL^−1^) was used to artificially infest the soil (Figure 7). Climatic factors, particularly precipitation, temperature, and soil humidity, probably directly influenced the short survival of the bacterium in the soil under field conditions. 

Studies with other bacteria of the genus *Xanthomonas* have shown variable results on the survival in the soil. *X. campestris* pv. *campestris* survived for only four to seven days in soil [33]. On the other hand, *X. vasicola* pv. *musacearum* survived for up to 20 days in the soil [39], while *X. citri* subsp. *malvacearum* survived for up to 50 days [40] and *X. citri* subsp. *citri* for 120 days on the soil surface [41]. 

Bacterial survival associated with crop residues is a common characteristic of several *Xanthomonas* spp. [42]. However, the survival period may vary depending on the bacterial species or pathovar [39,43,44]. Soil moisture has been considered a determinant of the survival of bacteria in crop residues [45,46]. Studies on *X. translucens* pv. *translucens* [47] and *X. euvesicatoria* [48] indicated that high temperatures can reduce the survival of these bacteria in crop residues. The experiments on the survival of Xvv in infected corn crop residues revealed a maximum survival period of 15 days for those maintained on the soil surface (Figure 7). The contrasting accumulated precipitation in the experimental periods (5.2 mm for the first experiment and up to 60 mm for the second experiment) did not influence the survival of the bacterium in the corn crop residues. 

In corn crop residues infected with the bacterium buried in the soil at a depth of 20 cm, viable Xvv cells were recovered only on the day the experiments were set up. However, the non-detection by isolation in selective culture medium may not be fully indicative of the absence of alive bacterial cells. The bacterium may be present at levels not detectable by conventional means. Ortiz-Castro et al. [13] examined the survival of Xvv associated with naturally contaminated corn crop residues under temperate climatic conditions in the USA. They did not recover viable bacteria cells from these residues maintained on the soil surface nor from those buried in the soil after six months. However, Xvv DNA was detected by qRT-PCR in the crop residues, but this technique does not allow to determine if a bacterium is alive or dead. Furthermore, it was observed that there were more bacterial cells in the crop residues kept on the soil surface compared to those buried in the soil. The results of the present study corroborate those obtained by Ortiz-Castro et al. [13]. Several studies indicated that the survival of plant pathogenic bacteria in crop residues maintained on the soil surface is longer compared to the survival in residues buried in the soil. It is worth mentioning studies carried out with *X. euvesicatoria* [48] and *X. citri* subsp. *citri* [41], as well as *Clavibacter michiganensis* subsp. *michiganensis* [49]. Similar results were obtained with *X. hortorum* pv. *vitians*. Fayette et al. [42] were able to recover viable cells of the bacterium from crop residues maintained on the soil surface for up to 30 days, while in samples buried in the soil it was not possible to recover viable bacterial cells.

The results obtained in this study showed that Xvv survives for a short period of time in soil and in crop residues under field conditions in humid subtropical climatic conditions. This short survival period suggests that soil and corn residues of previous crops would not be highly important sources of primary inoculum for leaf streak disease development in subsequent corn cultivation. This importance may be even reduced, for instance, in the case of a corn–soybean succession, as there is a fallow period between the corn crops. Further, soybean is not a host plant for Xvv [50]. However, it would be important to remove weeds and volunteer corn plants, potential hosts of the bacterium, as they can serve as reservoirs of Xvv [50], and to bury corn crop residues in the soil to accelerate decomposition and to have a corn succession with non-host species of the bacterium. 

## Figures and Tables

**Table 1:** Physical characteristics of the soils used in the study of the survival of *Xanthomonas vasicola* pv. *vasculorum* under controlled conditions.

Soil Type	Collection Site	Characteristics		
		Clay	Silte	Sandy
		(%)	(%)	(%)
Clayey	Londrina, PR	76	14	10
Sandy	Guairaçá, PR	9	2	89

**Table 2:** Chemical characteristics of the soils used in the study of the survival of *Xanthomonas vasicola* pv. *vasculorum* under controlled conditions.

Soil Type	pH	P	C	Al^3+^	H+Al	Ca^2+^	Mg^2+^	K^+^	SB	T	V	m
	(CaCl_2_ 0.01 M)	(mg dm^−3^)	(g dm^−3^)	(cmol_c_dm^−3^ of Soil)							(%)	
Clayey	5.00	92.70	16.44	0.04	5.76	5.40	1.85	0.68	7.93	13.69	57.92	0.50
Sandy	4.50	7.20	4.71	0.21	2.73	0.55	0.24	0.07	0.86	3.59	23.95	19.62

P and K: Mehlich I; C: Walkley—Black; Ca^2+^, Mg^2+^, Al^3+^: KCl 1 mol L^−1^; SB: sum of bases; T: cation exchange capacity; V: base saturation; m: aluminum saturation.

**Table 3:** Meteorological data at the IDR-Paraná Londrina Research Station, Londrina, PR. Brazil, during the experimental period in the years of 2020 and 2021 (I, II, III and IV experiments).

Day	Experimental Period																							
	I						II						III						IV					
	18 November through 24 November 2020						8 February through 14 February 2021						24 February through 2 March 2021						May 03 through May 09 2021					
	Tave (°C)	Tmax (°C)	Tmin (°C)	Rain (mm)	RH (%)	SM (%)	Tave (°C)	Tmax (°C)	Tmin (°C)	Rain (mm)	RH (%)	SM (%)	Tave (°C)	Tmax (°C)	Tmin (°C)	Rain (mm)	RH (%)	SM (%)	Tave (°C)	Tmax (°C)	Tmin (°C)	Rain (mm)	RH (%)	SM (%)
0	19.2	25	17.5	30.6	95	90	22.8	28.8	16.8	0	64	18	25.1	34.2	20.1	0.2	75	16	21.5	29	14.9	0	64	3
1	21.4	28.7	17.1	0.2	77	33	22.2	27.7	16.6	0	72	34	23.5	31.4	18.7	4.2	84	20	22	30.2	14.5	0	59	22
2	21.5	29.2	14.7	0	63	33	23.5	30.9	17.6	0	69	21	24.1	31.3	19.5	0	73	16	22.7	30.6	16.1	0	52	14
3	22.8	30.5	15.3	0	60	27	20.7	24.4	18.3	12.4	92	100	22.9	30.5	17.9	11	79	30	21.3	29.7	14.5	0	63	5
4	23.6	32.3	16.2	0	58	25	21.2	25.9	17.1	0.2	90	80	21.6	25.8	19.8	13.8	90	95	17.4	22	14.5	0	81	5
5	23.9	31.3	16.9	0	52	20	23.6	31.1	17.9	0	83	60	21.5	29.6	18.6	5.2	92	95	17.7	25.3	12	0	76	0
6	25.4	33.6	16.9	0	53	18	21.6	25.9	18	32.8	91	100	22.6	30.8	19.1	0.2	89	90	19.1	26.9	11.8	0	70	0
Mean	22.7	30.1	16.4	4.4	65.4	35.1	22.2	27.8	17.5	6.5	80.1	59.0	23.0	30.5	19.1	4.9	83.1	51.7	20.2	27.7	14.0	0.0	66.4	7.0

Tave: daily average air temperature, Tmax: maximum air temperature; Tmin: minimum air temperature; Rain: rainfall; RH: relative humidity; and SM: soil moisture.

## Data Availability

All data generated and analyzed during this study are presented in the published version of this article.
